# The Melanopic Sensitivity Function Accounts for Melanopsin-Driven Responses in Mice under Diverse Lighting Conditions

**DOI:** 10.1371/journal.pone.0053583

**Published:** 2013-01-03

**Authors:** Timothy M. Brown, Annette E. Allen, Jazi al-Enezi, Jonathan Wynne, Luc Schlangen, Vanja Hommes, Robert J. Lucas

**Affiliations:** 1 Faculty of Life Sciences, University of Manchester, Manchester, United Kingdom; 2 Philips Lighting, Eindhoven, The Netherlands; 3 Philips Consumer Lifestyle, Drachten, The Netherlands; Dalhousie University, Canada

## Abstract

In addition to rods and cones, photoreception in mammals extends to a third retinal cell type expressing the photopigment melanopsin. The influences of this novel opsin are widespread, ranging from pupillary and circadian responses to brightness perception, yet established approaches to quantifying the biological effects of light do not adequately account for melanopsin sensitivity. We have recently proposed a novel metric, the melanopic sensitivity function (V^Z^λ), to address this deficiency. Here, we further validate this new measure with a variety of tests based on potential barriers to its applicability identified in the literature or relating to obvious practical benefits. Using electrophysiogical approaches and pupillometry, initially in rodless+coneless mice, our data demonstrate that under a very wide range of different conditions (including switching between stimuli with highly divergent spectral content) the V^Z^λ function provides an accurate prediction of the sensitivity of melanopsin-dependent responses. We further show that V^Z^λ provides the best available description of the spectral sensitivity of at least one aspect of the visual response in mice with functional rods and cones: tonic firing activity in the lateral geniculate nuclei. Together, these data establish V^Z^λ as an important new approach for light measurement with widespread practical utility.

## Introduction

Light has a profound impact on mammalian behaviour and physiology. In order to effectively study or control such processes, it is essential to quantify light in a biologically meaningful way. The key to this challenge is the appreciation that biological photoreceptors are not equally sensitive to all wavelengths of light. So-called photometric measurement systems have been developed to meet this challenge. These employ filters to weight light energy across the spectrum according to the sensitivity of the physiological process under consideration. They provide the origin for the SI base unit Cd (and its derivatives lumen and lux) used to describe effective light exposure for human cone- (photopic) and rod- (scotopic) based vision.

The discovery of a new mammalian photoreceptor, melanopsin [1], [2], poses a new challenge for light measurement. It has been known for some time that melanopsin-based photoreception is a critical component of accessory visual responses such as circadian photoentrainment and pupillary reflexes [3], [4]. However, emerging evidence also implicates melanopsin in more conventional visual pathways including brightness perception [5]–[7]. Together, this work places renewed emphasis on defining appropriate photometric measures that account for light's impact on melanopsin.

**Figure 1:**
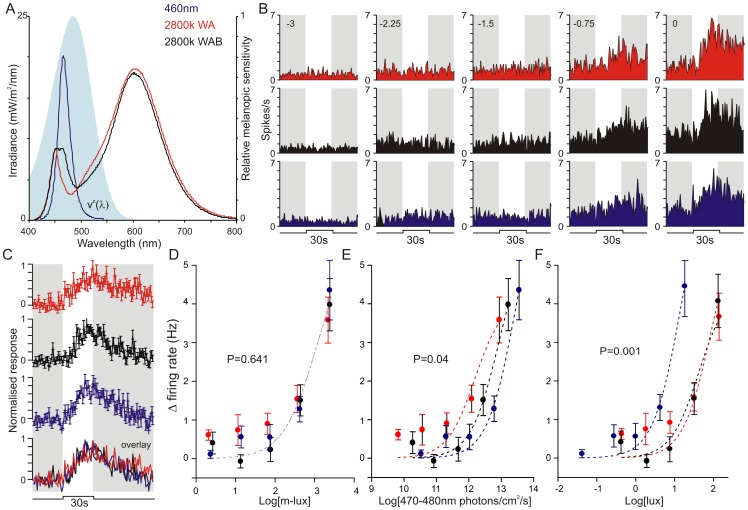
The melanopic sensitivity function accounts for the spectral sensitivity of LGN responses in rodless/coneless mice. (**A**) Spectral profile of the 3 test stimuli and the melanopic sensitivity function (V^z^λ: shaded area). (**B**) Example response of an *rd/rd cl* LGN neuron to the three test stimuli presented at a range of different irradiances (numbers above traces indicate log light intensity relative to the maximum achievable: 3.4 log m-lux). (**C**) Mean ± SEM responses to the three stimuli (and below in overlay) at maximum irradiance (n = 30 cells; each unit's response normalised to the largest change in firing rate across all stimuli). (**D–F**) Irradiance response relationship for *rd/rd cl* LGN responses to the three stimuli. A single curve best explains all the data (F-test) when irradiances are expressed in melanopic lux (**D**; P = 0.641), but not number of photons between 470–480 nm (**E**; P = 0.04), photopic lux (**F**; P = 0.001), total photons or total optical power (not shown).

Melanopsin's spectral sensitivity differs from that of rods and cones making established photometric measures inappropriate for quantifying effective light exposure for this new photoreceptor. We have recently proposed a solution to this problem in which ‘melanopic’ illuminance (m-lux) is estimated by weighting irradiance at each wavelength according to a sensitivity function V^Z^λ (Fig. 1A;[8]). V^Z^λ is based upon analytical action spectra in a number of species indicating that melanopsin's sensitivity can be approximated by the absorbance spectrum of an opsin:vitamin A photopigment with maximal absorption at 480 nm, corrected for prereceptoral filtering. To date, we have validated this approach to quantifying ‘melanopic’ illuminance by confirming that it accurately predicts the sensitivity of rodless+coneless mice (*rd/rd cl*; which rely solely upon melanopsin for photoreception) to an array of polychromatic lights. Here we set out to further test the utility of the V^Z^λ function for predicting melanopsin-dependent light responses under a wider array of conditions.

We started by exploring a recent suggestion that melanopsin-influenced physiological responses are disproportionately reliant upon 470–480 nm light [9]. Those data argue that V^Z^λ would provide a poor prediction of melanopsin responses to polychromatic lights that are very divergent in that part of the spectrum. We therefore set out to test that prediction using *rd/rd cl* mice.

Melanopsin has been proposed to have an intrinsic light-dependent bleach recovery mechanism whose spectral sensitivity is long wavelength shifted with respect to V^Z^λ [10], [11]. As such a mechanism is not accounted for in the proposed melanopic function, it has the potential to greatly limit the utility of this approach (for discussion see Methods). Our published data indicate that any such effect does not preclude using V^Z^λ to predict melanopsin's sensitivity to various polychromatic lights [8]. However, the photoregeneration process may have a particular impact upon melanopsin's sensitivity when there is temporal modulation in the spectral content of incident light. We therefore used *rd/rd cl* mice to test whether there were aspects of the melanopsin-driven response to changes in spectral composition that could not be predicted by V^Z^λ.

We next tested the utility of V^Z^λ in one of the most sophisticated applications of photometry – the generation of metamers. Metamers are spectrally distinct lights calculated to have the same photometric magnitude and therefore to appear indistinguishable for a particular visual process. They are a widely used tool in vision research and an important concept in engineering, providing, for example, the basis of using red, green and blue channels in visual displays to recreate the wide array of colour percepts induced by much more complex spectral distributions in the real world. We therefore set out to determine whether the V^Z^λ function could be used to design metamers for the melanopsin system.

A big advantage of the ‘melanopsin only’ *rd/rd cl* mice we have used thus far to test the V^Z^λ function is that these animals allow the spectral response properties of melanopsin to be studied in its native environment but isolated from any contaminating effects of rod or cone input. However, aspects of the light response that rely at least largely (and maybe wholly) upon melanopsin have been described also in animals with functional rods and cones [6], [12]–[18]. As the melanopic function is only really valuable if it predicts such events in visually intact organisms, our final experiment aimed to determine whether this was the case.

## Methods

### Ethics

All animal use received approval from the University of Manchester ethical review process committee and was in accordance with the Animals, Scientific Procedures, Act of 1986 (UK).

### Animals

Animals were housed under a 12-hour dark/light cycle environment at a temperature of 22°C with food and water ad libitum.

### In Vivo Neurophysiology

Visually-evoked neural activity was recorded as described previously [19], [20]. In brief, urethane (1.6 g/kg) anaesthetised adult male mice (80–160 days) were prepared for sterotaxic surgery and insertion of multielectrode arrays. Recording probes (A4X8-5 mm-50-200-413; Neuronexus, MI, USA) consisting of 4 shanks (spaced 200 μm), each with 8 recordings sites (spaced 50 μm) were then lowered to the level of the LGN or OPN according to stereotaxic coordinates. Probe placements were verified *post hoc* via histology. Neural signals were acquired using a Recorder64 system (Plexon, TX, USA), amplified (x3000), highpass filtered at (300 Hz) and digitized (40 kHz). Multiunit spikes were saved as timestamped waveforms and individual unit activity isolated offline via principle component-based spike sorting (Offline sorter, Plexon, TX, USA). During the experiments, adequate depth of anaesthesia was assessed by the absence of a hind-paw pinch reflex and additional doses of urethane (0.16 g/kg) applied as necessary. At the end of the experiments animals were culled by cervical dislocation.

### Pupillometry

Consensual pupil responses were monitored as described previously in unanaesthetised and gently restrained mice [8]. Full field visual stimuli were delivered to the right eye, previously dilated by topical atropine (1%), and infrared (>900 nm) video monitoring used to track pupil size in the left eye. Analysis of pupil area was performed offline using custom software.

### Light generation and measurement

For Neurophysiological experiements, visual stimuli were delivered via one of two custom built systems, each allowing us to combine light from up to 4 different LED sources. The first of these (Philips Consumer Lifestyle, Drachten, Netherlands), comprised one white (2800 k) and 3 chromatic LED sources (λmax: 467 nm, 598 nm, 637 nm; half bandwidth: 25, 70 and 18 nm respectively), combined in an integrating sphere and delivered to the subject via a 0.25” diameter quartz fibre optic light guide (Edmund optics, York, UK) placed ∼1 mm from the eye contralateral to the recording electrode. The second system (Cairn Research Ltd., Kent, UK) consisted of 4 bandpass filtered LED sources (λmax: 368 nm, 453 nm, 595 nm, 629 nm; half bandwidth all ∼15 nm) combined via dichroic mirrors and focused onto a diffuser (5 mm diam.) positioned ∼1 mm from the contralateral eye. For both systems, LED intensities were controlled independently via a PC running LabView 8.6 (National instruments, Berkshire, UK).

For pupillometry experiments, test stimuli were generated via a Xe-arc source (Cairn Research Ltd.). Intensity was regulated via neutral density filters and spectral composition via a 500 nm shortpass filter (Edmund optics) as appropriate. Stimulus timing was regulated by an electronic shutter (Uniblitz, NY, USA) and light was delivered via a quartz fiber optic to an integrating sphere positioned over the eye. Where appropriate, light pre-treatment was applied via a LED source (λmax: 498 or 644 nm; half bandwidth <37 nm; Philips Lighting) in a bespoke chamber with full internal reflectance. In other experiments, flickering stimuli (1–100 Hz) were generated by gating output from the Xe-arc source using a motorised ‘chopper’ wheel (Philips Lighting), which allowed for an open light path over a fixed 7.1% of the duration of each flicker cycle. In these experiments, a dual branch fiber optic was used to combine flickering stimuli with temporally uniform background lights (generated by a second Xe-arc source with appropriate interference filters).

All light measurements were performed using a calibrated spectroradiometer fitted with a cosine diffuser (Bentham instruments, Reading, UK) and calculations performed as described previously [8].

### Methodological considerations

There are an infinite range of conditions under which the melanopic function could be tested. Here we have focused on a selection based upon potential problems identified in the literature (addressing the possibility that light around 480 nm is especially significant, or that melanopsin may have responses to temporal modulations in spectral content unpredicted by V^Z^λ), or relating to obvious practical benefits (demonstrating its utility for visually intact animals and in designing metamers).

In terms of experimental design, the issue of temporal modulations in spectral content provides the biggest challenge – what wavelengths, irradiances and timescales have the best chance of revealing deviations in the melanopsin response from that predicted by V^Z^λ? We based our choice on the model proposed by Mure et al. [10], in which melanopsin is proposed to have two stable states: the inactive rhodopsin state (R; λmax = 480 nm), and its signalling active photoproduct metarhodopsin (M; λmax = 587 nm) which, in turn, reverts to the R state upon absorption of a second photon. This implies that the fraction of melanopsin in M vs. R states is dependent upon the spectral composition of incident light and that longer wavelengths shift the equilibrium in favour of the R state. This model was introduced as an explanation for evidence that melanopsin is photosensitised by prior exposure to red light, and a primary goal of these experiments was to determine whether such an event limits the utility of V^Z^λ in predicting the melanopsin response. Since, most of the data supporting this model derives from pupillary data [10], [11], we primarily addressed this goal by investigating pupil sensitivity.

Given the parameters of the model, the relative sensitivity of R and M pigments is greatest at long wavelengths, giving the largest fractional concentration of R following pretreatment with ‘red’ light (>650 nm). However, the rate at which this equilibrium is approached is dependent upon effective light intensity (and therefore is inversely proportional to wavelength for stimuli > the proposed M state λmax at 587 nm). As a compromise between these two imperatives, we chose conditioning stimuli in the range 600–650 nm to optimise generation of the R isoform and compared these against stimuli that should bias the equilibrium towards the M isoform (λmax<500 nm). In deciding the irradiance of these conditioning or background lights, we were mindful of the need to control for photoreceptor light adaptation. Accordingly we matched these stimuli to provide the same melanopic luminance (and therefore to be equally effective in driving the R to M isomerisation) and to be below threshold for evoking measurable physiological responses (0.4 m-lux). Determining sensitivity under these circumstances then involves recording responses evoked by a probe stimulus optimised to photoactivate the R pigment state. Nonetheless, as this probe stimulus will in itself shift the fractional concentration of M and R states, any effect of the background/pretreatment on sensitivity will be most apparent in responses to short duration flashes and these were used wherever possible.

A second implication of this model is that the lifetime of the M pigment should be dependent upon its rate of photon absorption. As it is the M state that is signalling active, this raises the possibility that long wavelength light could reduce melanopsin sensitivity by curtailing the lifetime of photoactivated melanopsin. Our published work with very red-shifted polychromatic lights in fact indicates that this is unlikely to be a significant concern [8]. This is consistent with the very low rate of photon absorption by the melanopsin system, which raises questions about how frequently this M to R transition occurs before the activated M is switched off by light-independent biochemical processes. Nevertheless, the impact of such a mechanism could in theory be especially marked for temporally modulated stimuli. At the extreme, one could envisage a stimulus of alternate ‘blue’ and ‘red’ lights in which the former switched melanopsin ‘on’ and the latter ‘off’.

Below, we test this latter possibility by assessing the impact of short (<500 nm) or long (>600 nm) wavelength background light on steady state pupil responses to 1Hz flickering probe stimuli of various irradiances. The greatest likelihood of seeing an effect under these conditions is when both probe and background lights are as bright as possible, maximising the likelihood of photoactivated M absorbing a second photon. We therefore used red lights at higher (5x) illuminances than in our pre-treatment studies. In addition to the data shown here, we tested a wide range of other conditions including: saturating 4 and 100 Hz flickering probe stimuli (<500 nm and unfiltered Xe-arc light; 669 and 1249 m-lux respectively) with various conditioning stimuli (500, 580, 620 and 650 nm monochromatic + >630 nm polychromatic; 10.1, 11.9, 13.3, 13.8 & 14.5 log photons/cm2/s respectively, all 0.2 m-lux); saturating 4Hz probe stimuli with brighter (x10) longwavelength background stimuli (>630 nm) and subsaturating 4 Hz probe stimuli (3 m-lux) with >630 nm background stimuli. In none of these situations did the appearance of the background light have any measurable effect on steady state pupil size.

## Results

### Case 1: An especial significance of light around 480nm?

To test whether melanopsin is more sensitive to light between 470 and 480 nm than predicted by V^Z^ **λ**, we established three spectrally distinct stimuli calculated to provide very similar melanopic illuminance (Fig. 1A, ∼3.4 log m-lux at peak intensity) but differing substantially in the number of photons over this part of the spectrum (12.4 to 13.6 log photons/cm^2^/s). We then compared electrophysiological responses to these three stimuli at a range of irradiances in one of the major targets of melanopsin ganglion cells, the intergeniculate leaflet and surrounding LGN [6], [7]. In order to isolate melanopsin-dependent responses, we performed these experiments in *rd/rd cl* mice, which are reliant upon melanopsin for their light response.

In total we isolated 30 light responsive single units from multiunit recordings in 6 *rd/rd cl* mice (Fig. 1B). Consistent with the predications of the melanopic sensitivity function, 30 s steps (from darkness) to the spectrally distinct stimuli were indistinguishable in magnitude or timecourse at each of the irradiances tested (Fig. 1C). Indeed, when we compared the intensity-dependence of firing activity elicited by these stimuli, we found that responses to all three could be explained by a single sigmoid function when illuminance was quantified in m-lux (Fig. 1D, F-test that curves different, P = 0.641), but not according to the number of photons at wavelengths between 470 and 480 nm (Fig. 1E, P = 0.04). Quantifying the data in photopic lux (Fig. 1F, P = 0.001), total photons, or total optical power also failed to adequately predict the sensitivity of LGN firing to the three stimuli (not shown, P = 0.003 and 0.008 respectively).

### Case 2: temporal modulation of spectral irradiance

It has been suggested that, thanks to its supposed bistability, prior light exposure could alter melanopsin sensitivity in ways unpredicted by the proposed melanopic function [10], [11]. As much of the evidence in support of that view comes from studies of the pupil light reflex, we next aimed to determine whether light pre-treatment influenced melanopsin-derived pupil responses with a spectral sensitivity different from that described by V^z^λ.

**Figure 2:**
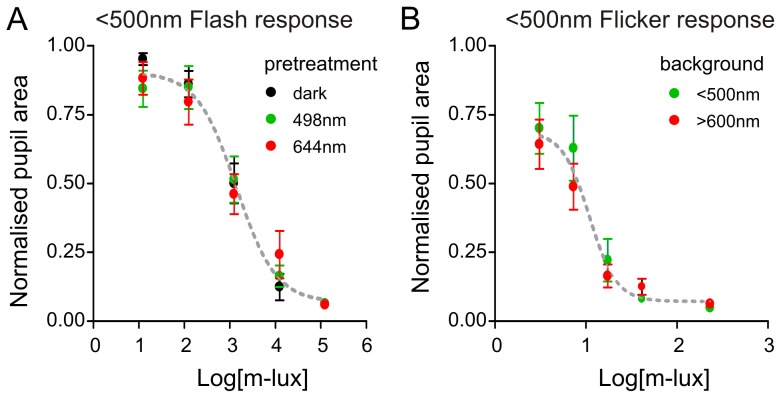
The melanopic sensitivity function accounts for the impact of long wavelength conditioning stimuli on pupillary responses in rodless/coneless mice. (**A**) Sensitivity of *rd/rd cl* pupil responses to shortwavelength (<500 nm) flash (100 ms) is not altered by 30 min pretreatment with short (498 nm) or long (644 nm) wavelength conditioning stimuli matched for melanopic illuminance (0.4 m-lux; F-test, P = 0.931). (**B**) Sensitivity of *rd/rd cl* pupil responses to <500 nm flicker (1 Hz; ON duration  = 71 ms), alternating with with short (<500 nm) or long (>600 nm) wavelength background illumination can be predicted when time-averaged irradiance is expressed in m-lux (F-test; P = 0.256).

Initially, we compared the pupil responses of *rd/rd cl* mice (n = 9) to a standard probe flash (100 ms Xe-arc source with 500 nm shortpass filter, for spectral profile see [8]) following either 1 h of dark or 30 min of pre-treatment with monochromatic 498 or 644 nm light. The melanopic illuminance of these two pre-treatments was matched (∼0.4 m-lux) and set to be sub-threshold for evoking a pupil response in their own right (to control for the possibility of photoreceptor adaptation). Under these conditions, the total number of photons in these two stimuli differed greatly (14.5 and 10.5 log photons/cm^2^/s for 644 and 498 nm respectively). According to the proposed model of melanopsin bistability (see Methods), any melanopsin in the M pigment state should be especially sensitive to the longer wavelength stimulus and, over 30 min of exposure, be regenerated to the photoreceptive R state, sensitising the system. Nevertheless, there was no significant effect of pre-treatment with either wavelength upon the magnitude or sensitivity of pupil responses elicited by the probe flash (Fig. 2A; F-test, P = 0.931).

An alternative potential effect of pigment bistability on melanopsin-dependent responses is that, by driving isomerisation of the M to R state, long wavelength stimuli could curtail the lifetime of photoactivated melanopsin. Such events are observed under extreme circumstances in invertebrate photoreceptors [21]. To explore the possible impact of such a mechanism we next compared the sensitivity of melanopsin-driven pupil responses in *rd/rd cl* mice to a flickering (1 Hz; flash duration 71 ms) short-wavelength light (<500 nm), alternating with either a short or long wavelength background (<500 or >600 nm, 11.3 and 14.9 log photons respectively). Once again, the background stimuli were matched in terms of melanopic illuminance, but were brighter than those used in the pre-treatment experiment above (2 m-lux) to maximise the chances of re-isomerising any active melanopsin within the relatively short interflash interval. According to the model of Mure et al. [10], the longer wavelength background should more effectively drive M to R isomerisation in melanopsin. If this in turn curtailed the lifetime of photoactivated M, we might expect a reduction in steady state pupil constriction compared to the shorter wavelength stimulus. In fact, we found pupil responses were indistinguishable between the two backgrounds (Fig. 2B; F-test, P = 0.257). In further experiments on the pupil light reflex we altered flicker frequency as well as the spectral composition of the flicker and background lights (see methods for conditions tested) but in no case did we observe an effect on pupil response that could not be predicted by the V^Z^λ function (not shown).

**Figure 3:**
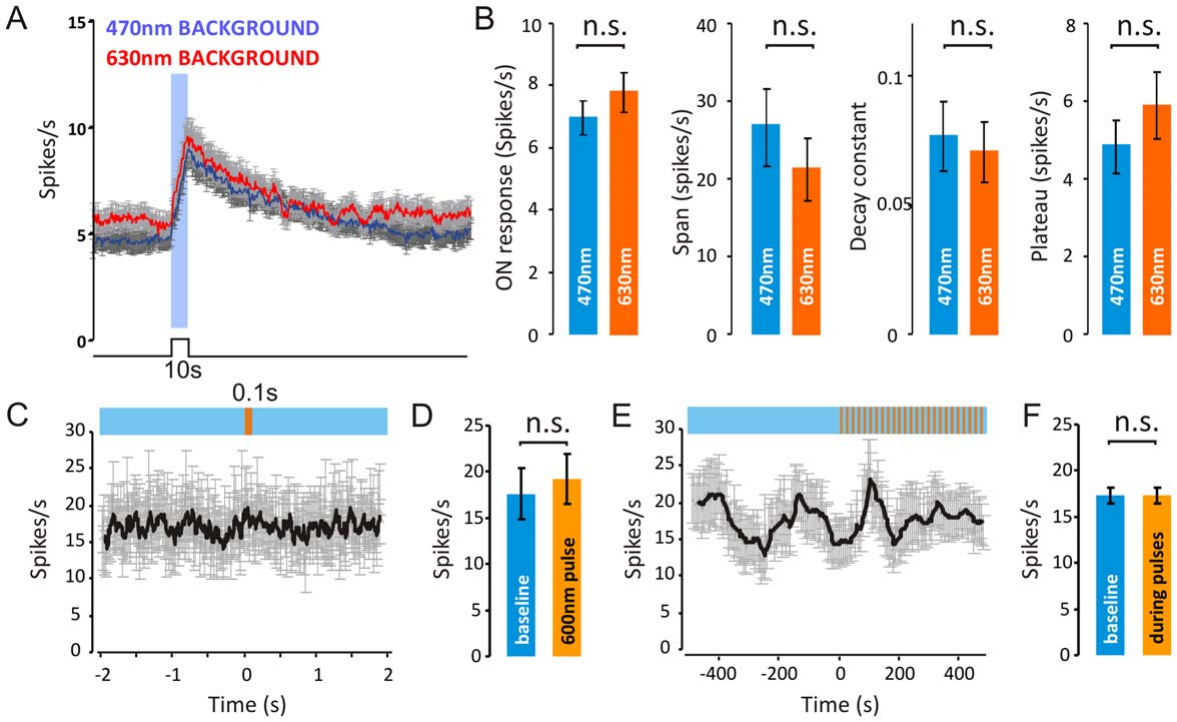
The temporal profile of melanopsin-dependent responses is equivalent under short and long wavelength adaptation. (**A**) Mean firing rate (± SEM) around a 10 s step of 470 nm (3.8 log m-lux; 10 repeats; interstimulus interval = 240 s) alternating with a background of 470 nm (blue line) or 630 nm (orange line). Backgrounds were calculated to provide −0.1 log m-lux, but differed in terms of total photons (10.6 and 14.6 log photons/m2/s, respectively). (**B**) Firing rate over the 10s stimulus was not significantly different between conditions (P = 0.34 two-sample T-test). (**C**) One-phase exponential decay curves were fitted to responses of each unit following light offset, and no significant difference was found in span, k-constant or plateau between the backgrounds (P = 0.40, P = 0.72 and P = 0.33, respectively, following two-sample T-test). (**C&D**) Long wavelength flashes (15.3 photons/cm2/s) calculated to produce a negligible change (∼1% increase) in melanopsin excitation drive no change in FR immediately following flash onset, and do not have any cumulative effects on ongoing responses (**E&F**).

To more directly test the possibility that the lifetime of photoactivated melanopsin is reduced under some backgrounds, we turned to electrophysiological recordings from the primary brain relay driving pupillary responses, the pretectal oliviary nucleus (PON). For these experiments we first investigated responses elicited by a series of 10 s steps to a subsaturating 470 nm stimulus (3.8 log m-lux; 10 repeats with inter stimulus interval  = 240 s) alternating with a background of either 470 nm or 630 nm light (10.6 and 14.6 log photons/m^2^/s, respectively) both calculated to provide ∼0.1 log m-lux. This paradigm thus allowed us to assess any wavelength-dependent effects of the background on firing induced by the 470 nm test stimulus, including the kinetics of response termination. In fact, we did not detect a significant influence of background wavelength on any aspect of the light response of over 131 PON units from 6 *rd/rd cl* mice (Fig. 3A&B).

As a final attempt to detect a change in either melanopsin sensitivity or deactivation kinetics associated with a long wavelength shift in light exposure, we applied a series of 600 nm pulses (100 ms, 20 s ISI; 2.2 log m-lux), superimposed on a melanopsin-activating 470 nm background (n = 18 light responsive PON units). These long wavelength pulses were calculated to provide only a 0.63% increase in melanopic illuminance. Consequently, if all aspects of melanopsin activity could be described by the V^Z^λ function, these flashes should drive little if any physiological response. On the other hand, if the longer wavelength stimulus curtailed the lifetime of photoactivated melanopsin, the flash should inhibit firing. Conversely, if the flash increased the rate of R pigment regeneration, it might be followed by a transient increase in firing. In fact, we found no significant alteration in firing during or after the 600 nm pulses (Fig. 3C–F).

### Case 3: Melanopic metamers

**Figure 4:**
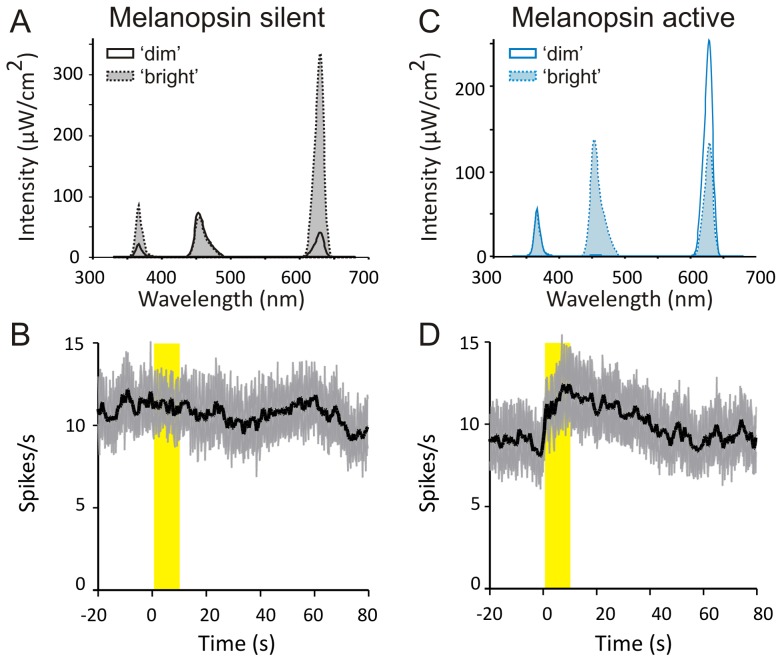
The melanopic sensitivity function accounts for OPN responses to spectrally modulated stimuli in rodless/coneless mice. (**A**) Spectral profile of stimuli that differ in irradiance but not melanopic illuminance (4-fold difference in total photons between ‘dim’ and ‘bright’), termed ‘melanopsin silent’. (**B**) Mean (± SEM) firing rate of 131 PON neurons to transitions between the two melanopsin silent stimuli. These transitions evoked no significant change in firing activity (paired t-test, P = 0.944). (**C**) Spectral profile of stimuli which differ substantially in melanopic illuminance (21-fold between dim and bright) but not total photons (<1% difference), termed ‘melanopsin active’. (**D**) Mean (± SEM) firing rate of PON neurons to transitions between the two melanopsin active stimuli. Transitions to the melanopsin ‘bright’ condition evoked a significant increase firing activity (paired t-test, P = 0.014, n = 131). Yellow bar in **C** & **D** indicates presentation of the ‘bright’ stimulus, with dim stimulus present at all other times.

As a final test of the ability of V^Z^λ to predict the sensitivity of melanopsin to spectrally modulated stimuli, we used this function to design spectrally divergent stimuli matched for melanopic illuminance (Fig. 4A; 4.8 log m-lux). If V^Z^λ successfully predicts the melanopsin response, then transitions between these stimuli should be undetectable for *rd/rd cl* mice. We found that this prediction was met. Thus, despite a substantial difference in photon flux between the two stimuli (15.7 to 16.3 log photons/cm^2^/s between ‘dim’ and ‘bright’ conditions), changing from one to the other induced no change in firing in the *rd/rd cl* PON (Fig. 4B). V^Z^λ should also enable us to design stimulus pairs matched for total photon flux but with divergent ability to activate melanopsin. We designed such a pair (Fig. 4C), whose photon flux was nearly identical (16.1 log/photons/cm^2^/s), but differing ∼21-fold in melanopic illuminance (3.7 to 5.0 log m-lux between ‘dim’ and ‘bright’ conditions). Consistent with our predictions, strong firing responses where elicited by transitions between elements of this pair (Fig. 4D). Similar results were obtained when the intensity of all stimuli were decreased by an order of magnitude (not shown).

### Case 4: Melanopic sensitivity in mice with functional rods/cones

We next sought to determine whether the V^Z^λ function provides a useful method of predicting aspects of the visual response of animals with functional rods and cones. We approached this by recording responses in the LGN where we have previously identified a strong melanopsin influence on electrophysiological responses [5], [6]. For these experiments, we used red cone knock-in mice (*Opn1mw^R^*; [22]). Cones in these mice develop and function normally, but those ordinarily expressing M-opsin instead express the red-shifted human L-opsin, and have a very clear difference in spectral sensitivity compared to melanopsin. This makes it much easier to identify response components that are especially reliant upon melanopsin [6], [12].

**Figure 5:**
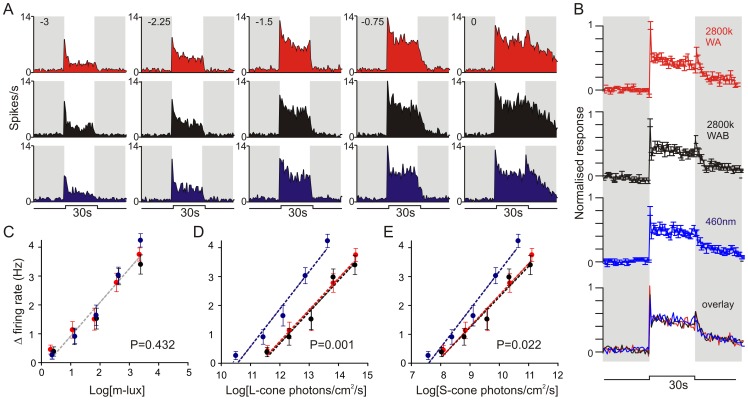
The melanopic sensitivity function accounts for the spectral sensitivity of tonic LGN firing activity in mice with functional rods/cones. (**A**) Example response of an *Opn1mw^R^* ‘sustained’ LGN neuron to the three test stimuli depicted in Fig. 1A at a range of irradiances (numbers above traces indicate log light intensity relative to the maximum achievable-3.4 log m-lux). (**B**) Mean ± SEM response to the three stimuli (and below in overlay) at maximum irradiance (n = 46 cells; each unit's response normalised to the largest change in firing rate across all stimuli). (**C–E**) Irradiance response relationship for *Opn1mw^R^* LGN sustained firing responses (20–30 s after stimulus onset) to the three stimuli. Sensitivity could be explained by a single linear function (F-test) when irradiances were expressed in melanopic lux (C; 0.432), but not in effective photon flux for L- or S-cones (**D** & **E**; P = 0.001 & 0.022 respectively).

From multiunit recordings in 6 *Opn1mw^R^* mice we identified 91 light responsive LGN units of which 46 showed sustained changes in firing under extended light exposure (Fig. 5A, B). Our earlier work, in this and other brain regions, indicates that the tonic component of such light responses is heavily dependent on melanopsin [6], [12], [20]. Accordingly we analysed the irradiance-dependence of firing activity evoked over the final 10 s of a 30 s light step. We found that this component of the response increased linearly over the range investigated, and that the coefficients of this relationship were not significantly different between the three stimuli when we quantified irradiances in m-lux (Fig. 5C, F-test, P = 0.432). By contrast, expressing irradiances with respect to their ability to activate either L– or UV-sensitive cone opsin (Fig. 5D, E; P = 0.001 and 0.022 respectively) did not account for the sensitivity of this response component to the three stimuli.

Given the similarity in spectral sensitivity of rods and melanopsin, it is perhaps unsurprising that responses were also similar when light exposure was expressed in scotopic luminance (not shown, F-test p = 0.202). However, the sensitivity range of this light response extends ∼2 orders of magnitude beyond the rod saturation point for such stimuli [19], [23]. Consequently, these data confirm that the melanopic function provides the most suitable available method for predicting this aspect of the LGN response to lights of diverse spectral content even in fully sighted animals.

## Discussion

The absence of an accepted approach to measuring light as experienced by melanopsin photoreceptors represents a barrier to comparing and replicating experimental findings obtained using different light sources, and to relating observations in the laboratory to lighting conditions in the field. We have previously proposed a simple method of calculating ‘melanopic’ illuminance based upon an approximation of melanopsin's spectral sensitivity (V^Z^λ). Here we set out to test this method under a wider array of conditions than those attempted in our first presentation of that method. Specifically, we tested whether the V^Z^λ function allows us to: 1.) predict melanopsin's sensitivity to polychromatic stimuli that are very divergent in the quantity of light in 460–480 nm wavelength range; 2.) predict melanopsin dependent aspects of the visual response in intact mice; 3.) predict melanopsin's response to temporal modulations in spectral content; and 4.) design melanopsin metamers. In all cases, the answer was ‘yes’. Data such as these can never exclude the possibility that limitations in the proposed method of calculating ‘melanopic’ illuminance would be revealed under different circumstances, or using more sensitive methods of measurement. However, taken together with our published validation of this approach for lights of divergent spectral quality [8], they provide confidence that it is suitable under most conditions. Moreover, these findings show that the proposed melanopic metric represents the best method currently available for predicting the sensitivity of melanopsin-driven responses in both retinally degenerate and visually intact animals.

The V^Z^λ function is based upon the spectral absorbance template for opsin photopigments and therefore assumes that sensitivity changes rather little over a wide range of wavelengths close to its peak [24]. It was surprising therefore that Rahman et al. [9] reported white light-driven endocrine/clock gene responses could be essentially abolished by selectively filtering a narrow range of wavelengths around the peak in melanopsin sensitivity (470–480 nm). Such an effect could be very useful from a lighting design perspective as it would be relatively easy to regulate this part of the spectrum without impacting photopic luminance or perceived colour. Unfortunately, our data do not support the view that melanopsin is anomalously sensitive to these wavelengths and therefore question whether the effect reported previously is a general feature of melanopsin-derived responses.

A substantial body of evidence suggests that, unlike other mammalian opsins, the photo-activated form of melanopsin (M-state) is stable and can be converted back to the inactive form (R-state) by absorption of a second photon of light [10], [11], [25], [26]. Many invertebrate opsins posses this property (bistability), with the spectral sensitivity of this reversal often long-wavelength shifted relative to the forward reaction [27]. Accordingly, there is evidence that pre-treatment with long-wavelength light can potentiate melanopsin responses in mice and humans [10], [11], [28] (although see [29], [30]).

While we did not set out to directly test whether melanopsin is bistable, we were concerned that such a mechanism might preclude the use of the melanopic sensitivity function under certain conditions. In particular we considered here two situations where melanopsin responses might be influenced by a wavelength-dependent pigment regeneration mechanism:

1. By determining the fractional content of R vs M state melanopsin, the regeneration mechanism could impact the efficiency of photon capture by the photoactivatable R state. Such an event has been invoked to explain evidence that melanopsin-driven responses in mice and humans are more sensitive following long wavelength exposure [10], [11]. Our experiments did not reveal any effect of pre-treatment with spectrally distinct light on melanopsin sensitivity that could not be predicted by the V^Z^λ function. This was the case both for measures of the pupil response following long-term (30 mins) pretreatment, and for electrophysiological responses in the PON induced by light steps alternating with spectrally distinct backgrounds.
2. Light absorbance by the photoactivated M-state could return it to the signalling-silent R state and therefore reduce the longevity of the melanopsin photoresponse. We tested for such an effect directly, by assessing the lifetime of the PON neuron firing response induced by steps of light presented against long vs short wavelength backgrounds matched for melanopic illuminance. Our failure to see any difference in this parameter under the two conditions indicates that, if the proposed regeneration mechanism does curtail M-state lifetime, the V^Z^λ function provides a reasonable estimate of the spectral sensitivity of such an event. Alternatively, events other than melanopsin kinetics may define the termination of the PON electrophysiological response under these conditions. Our further observations that PON firing activity and the sensitivity of pupil responses to flickering light are not influenced by spectrally divergent background lights go some way to addressing the latter concern. Moreover, as such flickering stimuli represent the only realistic way in which temporally modulated changes in spectral composition could be used to control melanopsin sensitivity in the field, our data imply that under most practical circumstances this proposed feature of melanopsin photobiology does not preclude use of the melanopic metric.

The protocols we employed here to explore the impact of melanopsin's proposed bistability were based primarily upon data indicating that the photoreversal/regeneration event is substantially long wavelength shifted. More recently, Matsuyama et al. [26], studying this process in purified protein in vitro, have suggested a much more modest difference in spectral sensitivity between the two forms of mouse melanopsin. Our data were not designed explicitly to distinguish between these two possibilities. If the two melanopsin states had similar spectral sensitivity that could certainly explain why melanopsin's light-dependent bleach recovery does not preclude the use of V^Z^λ to predict its sensitivity. However, it also remains possible that this event does not impact on the availability of photosensitive melanopsin or the lifetime of photoactivated melanopsin sufficiently to have a measurable effect on sensitivity under the circumstances studied here. Either explanation is comforting for the continued applicability of the proposed melanopic measure.

Finally, our demonstration that V^Z^λ provides the best available prediction of tonic light-driven firing activity in the LGN of mice with intact rods and cones is particularly important, given recent data indicating that melanopsin influences brightness perception in both mice and humans [5]. Those findings are themselves consistent with earlier data indicating that photopic luminance, the current standard used to quantify light intensity as experienced by the human visual system, does not always account for perceived brightness [31]–[35]. Together, then, these data suggest that V^Z^λ could have more widespread practical utility as a metric for quantifying apparent brightness, next to it's role as an experimental tool. Indeed, incorporating melanopic luminance as a design consideration, alongside established photometric measures, could facilitate a range of applications such as ambient lighting and visual display units with optimal perceived brightness, biological efficacy and minimal energy expenditure.
